# Canonical and Non-canonical Inflammasome Activation by Outer Membrane Vesicles Derived From *Bordetella pertussis*

**DOI:** 10.3389/fimmu.2020.01879

**Published:** 2020-08-20

**Authors:** Maia L. Elizagaray, Marco Túlio R. Gomes, Erika S. Guimaraes, Martín Rumbo, Daniela F. Hozbor, Sergio C. Oliveira, Griselda Moreno

**Affiliations:** ^1^Instituto de Estudios Inmunológicos y Fisiopatológicos (IIFP), Facultad de Ciencias Exactas UNLP CONICET, La Plata, Argentina; ^2^Departamento de Bioquímica e Imunologia, Instituto de Ciências Biológicas, Universidade Federal de Minas Gerais, Belo Horizonte, Brazil; ^3^Programa de Pós-Graduação em Genética, Instituto de Ciências Biológicas, Universidade Federal de Minas Gerais, Belo Horizonte, Brazil; ^4^Laboratorio VacSal, Facultad de Ciencias Exactas, Instituto de Biotecnología y Biología Molecular (IBBM), CCT-CONICET La Plata, Universidad Nacional de La Plata, La Plata, Argentina

**Keywords:** inflammasome, caspases 1/11, *Bordetella pertussis*, outer membrane vesicles, LOS

## Abstract

Outer Membrane Vesicles (OMVs) derived from different Gram-negative bacteria have been proposed as an attractive vaccine platform because of their own immunogenic adjuvant properties. Pertussis or whooping cough is a highly contagious vaccine-preventable respiratory disease that resurged during the last decades in many countries. In response to the epidemiological situation, new boosters have been incorporated into vaccination schedules worldwide and new vaccine candidates have started to be designed. Particularly, our group designed a new pertussis vaccine candidate based on OMVs derived from *Bordetella pertussis* (BpOMVs). To continue with the characterization of the immune response induced by our OMV based vaccine candidate, this work aimed to investigate the ability of OMVs to activate the inflammasome pathway in macrophages. We observed that NLRP3, caspase-1/11, and gasdermin-D (GSDMD) are involved in inflammasome activation by BpOMVs. Moreover, we demonstrated that BpOMVs as well as transfected *B. pertussis* lipooligosaccharide (BpLOS) induce caspase-11 (Casp11) and guanylate-binding proteins (GBPs) dependent non-canonical inflammasome activation. Our results elucidate the mechanism by which BpOMVs trigger one central pathway of the innate response activation that is expected to skew the adaptive immune response elicited by BpOMVs vaccination.

## Introduction

Outer membrane vesicles (OMVs) are nano-structures that are released spontaneously from the cell envelope of Gram-negative bacteria (1). OMVs are typically 50–200 nm in diameter, and are produced during different growth phases and in several environmental conditions studied to date (2). OMVs from some microorganisms are also able to deliver a diversity of virulence factors, including toxins, adhesins, and cell wall components such as peptidoglycan and lipopolysaccharide (LPS), immunomodulatory molecules directly into host cells during infection (3–8). Owing to their adaptability as a delivery vehicle, the contributions of OMVs to bacterial fitness are diverse, but there is increasing interest on their role in host colonization and disease pathogenesis (9, 10). These features and the intrinsic adjuvant capacity render OMVs as promising candidates for vaccine development against bacterial infections (11, 12). Despite their prophylactic potential there are few studies exploring the direct effects of OMVs on monocytes/macrophages and inflammasome activation. Inflammasomes are multiprotein complexes that assemble in the cytosol of different epithelial and immune cells, particularly in macrophages, and involve different sensors and caspases (13). Using the *Bordetella pertussis* (etiologic agent of the respiratory disease named pertussis) *in vitro* infection model there is evidence of inflammasome activation in human and murine macrophages and, the protective IL-1β response was demonstrated to be caspase-1-independent (14, 15). IL-1β production in mouse dendritic cells is triggered by *B. pertussis* virulence factor adenylate cyclase, through NLRP3 cytosolic sensor and consequent activation of caspase-1. This inflammasome activation leads to an antigen specific Th17 response in the murine infection model (16, 17). Activation of the inflammatory caspase-1 by detection of Danger-Associated Molecular Patterns (DAMPs) or Pathogen Associated Molecular Patterns (PAMPs) by different cytosolic inflammasome sensors triggers the processing of the pro-forms of interleukin IL-1β and IL-18 with their subsequent release to the extracellular milieu. This capase-1 dependent process is termed as “canonical” inflammasome activation. A “non-canonical” inflammasome activation has been described as a pathway that is dependent on caspase-11 (in mice) or caspase-4/5 (in humans). These inflammatory caspases can sense intracellular LPS from gram-negative bacteria and together with caspase-1 can cleave the pore forming protein gasdermin-D (GSDMD). GSDMD permeabilizes the cell membrane and may trigger pyroptosis, a form of inflammatory programmed cell death. However, release of IL-1β and IL-1α cytokines is feasible in the absence of cell lysis through GSDMD pores, likewise the release of high mobility group box 1 (HMGB1) alarmin molecules and even the entire inflammasome complexes including caspase-1 (18–20). The inflammatory cytokine IL-1β is an endogenous mediator that modulate immunity in the host and is involved in the induction of a Th17/Th1 profile *in vivo* in response to pathogens (21).

Pertussis or whooping cough is a highly contagious vaccine-preventable respiratory disease. Infant immunization programs with pertussis vaccines have been vastly successful in preventing the severe disease (22). However, in recent years the disease has reemerged in communities/states/countries with a striking high number of cases (23, 24). Multiple factors that could explicate the resurgence of the disease have been proposed, most of them related to present-day vaccines: waning vaccine-induced immunity, the switch from whole cell vaccines (wP) to acellular vaccines (aP) and pathogen adaptation (25–27). We have developed a new acellular anti-pertussis vaccine candidate based on OMVs derived from *B. pertussis* (BpOMVs) that has similarities with the main relevant properties of current aP vaccines in terms of biosafety and those of wP vaccines in terms of immunogenicity and protective capacity (28, 29).

In the present study, we show that BpOMVs are capable of triggering inflammasome activation in murine and in human macrophage cells. We characterized the mechanism by which BpOMVs trigger inflammasome activation, showing that IL-1β production by BpOMVs is dependent on canonical NLRP3 inflammasome and the adaptor molecule ASC [Apoptosis-associated speck-like protein containing a caspase recruitment domain (CARD)]. Moreover, we demonstrated that BpOMVs as well as transfected *Bordetella pertussis* lipooligosaccharide (BpLOS) induces caspase-11 (Casp11) dependent non-canonical inflammasome activation and guanylate-binding proteins (GBP) contained in mouse chromosome 3 (GBPchr3) expression in murine macrophages (BMDM).

## Materials

### Materials and Methods

#### Isolation of Outer Membrane Vesicles (OMVs)

OMVs were isolated from bacterial cells as previously described (28, 30). Briefly, culture samples from the decelerating growth phase were centrifuged at 10,000 × g for 20 min at 4°C and the bacterial pellet obtained was resuspended in 20 mM Tris–HCl, 2 mM EDTA pH 8.5 (TE buffer). Five milliliters of TE buffer were used to resuspend approximately 1 g (wet weight) of bacteria. The suspension was sonicated in cool water for 20 min. After two centrifugations at 10,000 × g for 20 min at 4°C, the supernatant was pelleted at 100,000 × g for 2 h at 4°C. This pellet was resuspended in 1.5% (w/v) deoxycholate (DOC) in TE buffer. Six milliliters of this suspension were added on 2 ml of sucrose 60% (w/v). After centrifugation at 100,000 × g for 2 h at 4°C, the OMV band was observed at TE/sucrose interphase. The OMVs were stored with glycerol 1% and sodium azide 0.001%? at 4°C. The samples obtained for all the *B. pertussis* strains used were negatively stained and then examined with an electron microscope.

#### Ethics Statement

All experiments involving animals were conducted in accordance with the Brazilian Federal Law number 11.794, which regulates the scientific use of animals in Brazil, the Institutional Animal Care and Use Committees (IACUC) guidelines, and the Animal Welfare Act and Regulations guidelines established by the American Veterinary Medical Association Panel on Euthanasia. Animals were fed, housed, and handled in strict agreement with these recommendations. All protocols were approved by the Committee for Ethics in Animal Experimentation (CETEA) at Universidade Federal de Minas Gerais UFMG under permit #165/2019. In Argentina, animals were fed, housed, and handled in strict agreement with the recommendations, protocols, and guidance of CICUAL (Institutional Committee for the Care and Use of Laboratory Animals) with the approved protocol #005-06-15 extended in validity until August 10, 2023.

#### Mice

In Brazil, wild-type C57BL/6 mice were purchased from the Federal University of Minas Gerais (UFMG). Nlrp3^−/−^ and Casp1/11^−/−^ were described previously and backcrossed to C57BL/6 mice for at least eight generations (31, 32). Casp11^−/−^, Gsdmd^−/−^, and Gbpchr3^−/−^ mice were generated in the C57BL/6 background (33–37). The animals were maintained at UFMG under 12 h cycles of light/dark and used at 6–9 week of age. In Argentina, wild type C57BL/6 mice were purchased from Laboratory of experimental animals, LAE- Faculty of Veterinary Sciences of the National University of La Plata (UNLP).

#### Generation of Bone Marrow Derived Macrophages (BMDM)

BM cells were obtained from femur and tibiae of *knockout* (KO) and *wild type* (WT) mice, and they were differentiated into BMDMs using a previously described protocol, with some modifications (38). Briefly, cells were seeded on 24-well plates at 5 × 105 cell/mL (day 0) and maintained in Dulbecco's Modified Eagle Medium- DMEM medium containing 10% FBS, 100 U/mL penicillin, 100 μg/mL streptomycin, and 20% L929-conditioned medium (LCCM), at 37°C in a 5% CO_2_ atmosphere for 7 days. On day 4 of incubation, the medium was fully replaced.

#### *In vitro* Stimulation of BMDMs

In all experiments, BMDMs were maintained in DMEM medium containing 10% FBS. Cells were stimulated with BpOMVs (800 ng/mL) or with free or transfected BpLOS [obtained and purified at our lab as previously described (39)] (800 ng/mL) in 0.5 mL DMEM + 10% FBS, previously incubated with indicated concentration of KCl for 1 h, where applicable. BpLOS transfection was carried out with FuGENE® HD Transfection Reagent (Promega®) according to manufacturer's specifications. Supernatants were collected at indicated times. Cells were then washed with PBS at room temperature, lysed with 25 μL/well M-PERR + 10 mM NaF, 1 mM sodium orthovanadate, and 1:100 protease inhibitors cocktail and collected in 1.5 mL tubes. Both supernatants and cell lysates were stored at −80°C until use.

#### Western Blot Analysis

BMDM culture supernatants were collected, and cells were lysed with M-PER Mammalian Protein Extraction Reagent (Thermo Fisher Scientific) supplemented with 1:100 protease inhibitor mixture (Sigma-Aldrich). Cell lysates and supernatants were subjected to SDS-PAGE analysis and western blotting. The proteins were resuspended in SDS containing loading buffer, separated on a 15% SDS-PAGE gel, and transferred to nitrocellulose membranes (Amersham Biosciences, Uppsala, Sweden) in transfer buffer (50 mM Tris, 40 mM glycine, 10% methanol). Membranes were blocked for 1 h in TBS with 0.1% Tween-20 containing 5% non-fat dry milk and incubated overnight with primary antibodies at 4°C. Primary Abs used included a mouse monoclonal against the p20 subunit of caspase-1 (Adipogen, San Diego, CA, USA) and the p17 subunit of IL-1β (Cell Signaling Technology, Danvers, MA) at a 1:1,000 dilution. Loading control blot was performed using mAb anti–β-actin (Cell Signaling Technology, Danvers, MA) at a 1:1,000 dilution. The membranes were washed three times for 5 min in TBS with 0.1% Tween 20 and incubated for 1 h at 25°C with the appropriate HRP-conjugated secondary Ab (Cell Signaling Technology, Danvers, MA) at a 1:1,000 dilution. Immunoreactive bands were visualized using Luminol chemiluminescent HRP substrate (Millipore) and analyzed using the ImageQuant TL Software (GE Healthcare, Buckinghamshire, United Kingdom).

#### RNA Isolation From BMDMs and Quantitative Real-Time PCR

Total RNA from stimulated BMDMs was isolated using the TRIzol® reagent, accordingly to manufacturer instructions. Reverse transcription of 1 μg from total RNA was performed using illustra^TM^ Ready-To-Go RT-PCR Beads (GE Healthcare, UK), following instructions of the manufacturer. Quantitative real-time PCR was performed using SYBR Green PCR master mix (Applied Biosystems, Foster City, CA) on a QuantStudio3 real-time PCR instrument (Applied Biosystems, Foster City, CA). The specific primers to amplify fragments corresponding to specific gene targets were used: β-actin, forward, 5′-GGCTGTATTCCCCTCCATCG-3′, reverse, 5′-CCAGTTGGTAACAATGCCATGT-3′; GBP1, forward, 5′-GAGTACTCTCTGGAAATGGCCTCAGAAA-3′, reverse, TAGATGAAGGTGCTGCTGAGGAGGACTG-3; GBP2, forward, 5′-CTGCACTATGTGACGGAGCTA-3′, reverse, 5′-CGG AATCGTCTACCCCACTC-3′; GBP3, forward, 5′-CTGACAGTAAATCTGGAAGCCAT-3′, reverse, 5′-CCGTCCTGCAAGACGATT CA-3′; GBP5, forward, 5′-CTGAACTCAGATTTTGTG CAGGA-3′, reverse, 5′-CATCGACATAAGTCAGCACCAG-3′; GBP7, forward, 5′-TCCTGTGTGCCTAGTGGAAAA-3′, reverse, 5′-CAAGCGGTTCATCAAGTAGGAT-3′. All data are presented as relative expression units after normalization to the β*-actin* gene. Measurements were conducted in triplicate.

#### Cell Line Stimulation

THP1-ASC-GFP (InvivoGen®) were maintained in RPMI medium containing 10% FBS, 100 U/mL penicillin, 100 μg/mL streptomycin at 37°C in a 5% CO_2_ atmosphere and plated at 3.6 × 10^5^ cells/well in 96-well plates. Cells were stimulated with BpOMVs (800 ng/mL). For positive control stimulus, cells were primed with ultrapure LPS from *E. coli* (Sigma-Aldrich) 1 μg/mL (LPS_*E*.*coli*_) for 3 h and transfected with bacterial plasmid cDNA (500 ng/mL). pcDNA was generated by cloning PCR-generated full-length cDNA from a random non-related gene. Transfection was carried out with Lipofectamine™ LTX Reagent with PLUS™ Reagent (Invitrogen) following manufacturer instructions. Supernatants were harvested and stored at −80°C until use and cells were prepared for microscopy analysis.

THP1-XBlue™-defMyD (InvivoGen®) were maintained in RPMI medium containing 10% FBS, 100 U/mL penicillin, 100 μg/mL streptomycin at 37°C in a 5% CO_2_ atmosphere and plated at 1.8 × 10^5^ cells/well in 96-well plates. Cells were stimulated with BpOMVs (800 ng/mL). For positive control stimulus, cells were stimulated with 50 ng/mL TNF-α, as manufacturer recommendations. Supernatants were harvested and stored at −80°C until use for cytokines and SEAP measurements.

#### Microscopy ASC-GFP Specks Observation

THP1-ASC-GFP cells were harvested after ON stimulation, washed with PBS and centrifugated. Nuclei were DAPI stained and 2% PFA fixation was performed. The number of ASC-GFP positive cells and localization of fluorescent ASC specks were determined using a Nikon Eclipse Ti Fluorescence Microscope and analyzed with ImageJ. Every experimental condition was carried out in triplicated and three pictures per well were taken. The relation between total cells and ASC-GFP specks was calculated. The result shown in this study is the output of three independent experiments.

#### Cytokine Determination in Supernatants by ELISA

Collected supernatants of stimulated BMDMs were thawed on the day of the assay and used for determination of mIL-1β, mIL-1α, mIL-12, and mTNF-α concentrations using Mouse DuoSet ELISA (R&D Systems), according to the manufacturer's specifications. hIL-1β and hIL-8 in supernatants of THP1 cells were measured using human OptEIA™ ELISA Set (BD), according to the manufacturer's specifications. hIL-18 in supernatants of THP1 cells were measured using human IL-18 ELISA (eBioscience), according to the manufacturer's specifications.

#### Statistical Analysis

All experiments were repeated at least three times with similar results. Graphs and data analysis were performed using GraphPad Prism 6 (GraphPad Software), using one-way ANOVA (Bonferroni *post-hoc* test) or Student's *t*-test (Tukey's *post-hoc* test). All quantitative data are expressed as mean ± SEM. A *P* < 0.05 (*p* ≤ 0.05) was considered statistically significant.

## Results

### BpOMVs Induce IL-1β Secretion in Unprimed Human and Murine Macrophages and Partially Requires Potassium Efflux

**Figure 1:**
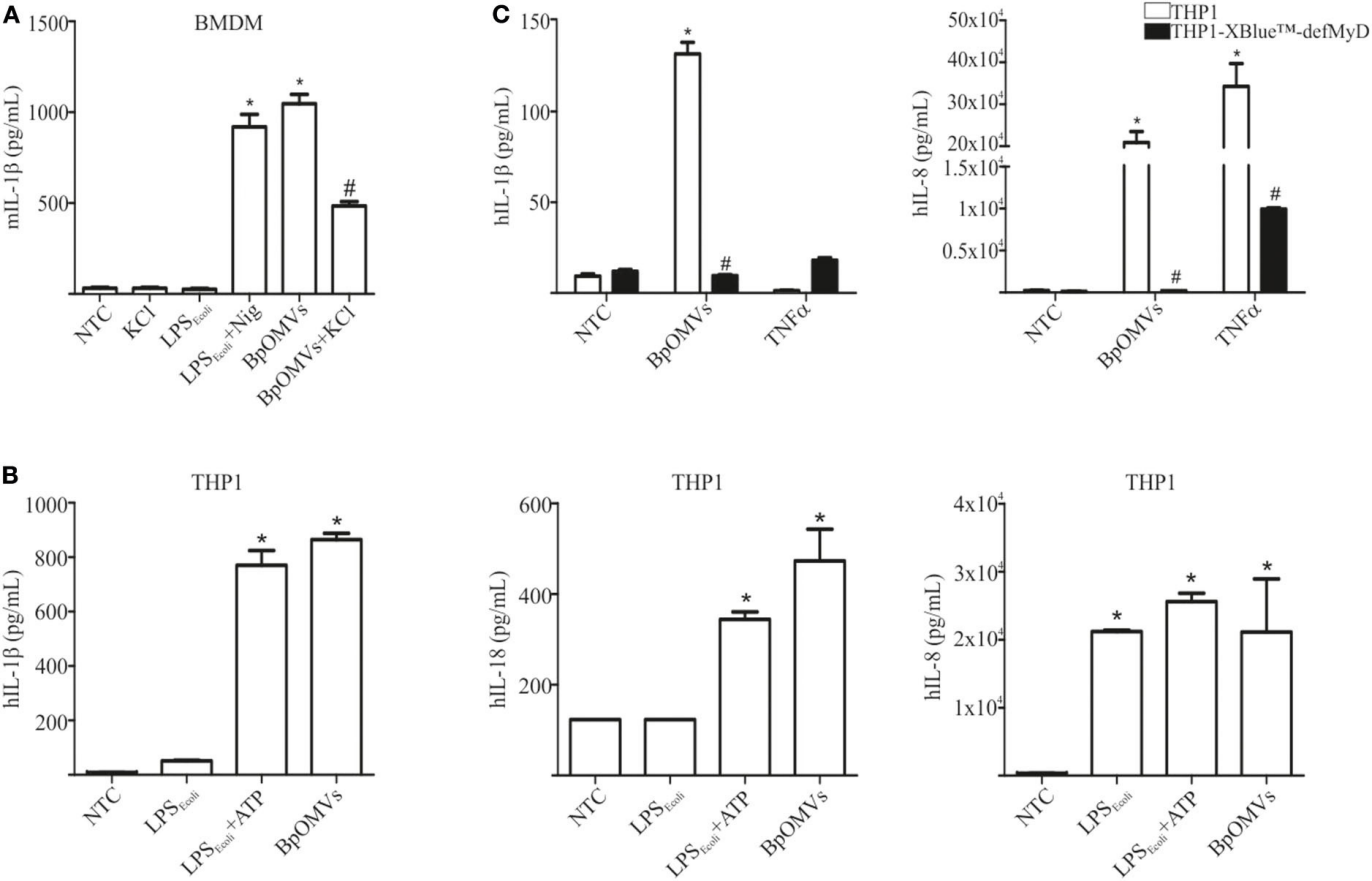
OMVs derived from *B. pertussis* (BpOMVs) triggers IL-1β in murine and human macrophages. **(A)** BMDM from C57BL/6 mice were stimulated ON with BpOMVs previously treated or not with 80 mM KCl for 30 min, and mIL-1β was measured in supernatants. As positive control, cells were primed with LPS_*E*.*coli*_ and treated with Nigericin for 3 h. **(B)** THP1 cells were stimulated ON with BpOMVs. As a positive control, cells were primed with LPS_*E*.*coli*_ for 3 h, then incubated ON with ATP. hIL-1β, hIL-18, and hIL-8 were measured in supernatants by ELISA. **(C)** THP1-XBlue-defMy88 cells were stimulated ON with BpOMVs. As a positive control cells were treated with TNF-α. *A result significantly different (*p* < 0.05) from NTC; ^#^a result significantly different from BpOMVs stimulation (*p* ≤ 0.05).

**Figure 2:**
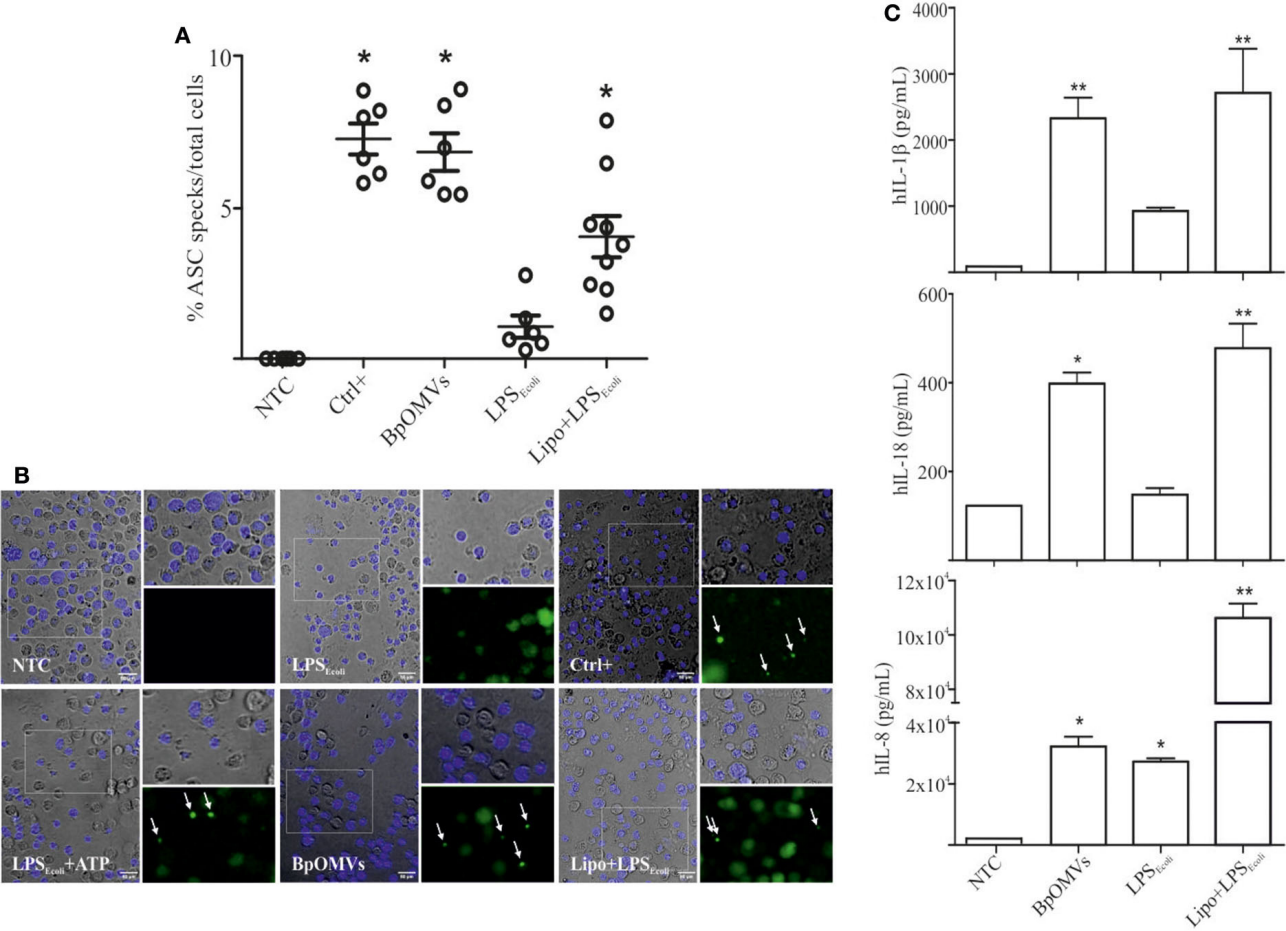
OMVs derived from *B. pertussis* (BpOMVs) triggers ASC speck formation in THP1-ASC-GFP cells. **(A)** THP1-ASC-GFP cells were stimulated ON with BpOMVs. Transfected LPS_*E*.*coli*_ was used as a positive control for NLRP3 pathway. Cells were primed with LPS_*E*.*coli*_ for 3 h, medium fully replaced and transfected with pcDNA ON as a positive control for optimal visualization of specks. GFP-ASC specks were counted and the relation to total cells calculated. Data on graph show a representative experiment. Three independent experiments were performed. Each point represents average number of total speck/total cells per field. Three fields of each treatment were counted (2 replicates/treatment). *A result significantly different (*p ≤ 0.05) to NTC **(B)**. Representative microscopy pictures from **(A)** are shown. White arrowheads indicate the ASC-GFP. **(C)** hIL-1β, hIL-18, and hIL-8 were measured in supernatants by ELISA. Data are represented as mean ± SEM of three replicates. *A result significantly different (*p ≤ 0.05 and **p ≤ 0.01) from their respective control (NTC or LPS_*E*.*coli*_, respectively).

As a first approach to investigate the induction of IL-1β secretion triggered by BpOMVs in BMDM and in a human macrophage cell line (THP1), we performed a dose-response assay to select the BpOMVs concentration to be used along the different experiments. IL-1β levels, LDH values, and morphological microscopy and flow cytometry analysis were performed in order to select conditions with minimal alterations on cell viability and significant cytokine secretion (data not shown). In all cases, primed and unprimed murine macrophages secreted significantly higher amounts of mIL-1β (Figure 1A and Supplementary Figure 1) in response to BpOMVs compared to control treatments (primed or NTC, respectively). mTNF-α and mIL-6 were used as surrogate readout of NF-κB pathway activation and their secretion was increased either by BpOMVs treatment as well as by priming with LPS_*E*.*coli*_, as expected (Supplementary Figure 1). Secretion of the proinflammatory cytokines IL-1β and IL-18 depends on the activation of inflammasome multiprotein platforms. Although different cytosolic sensor proteins may trigger inflammasome assembly, the NLRP3 complex has been the most studied to date (40). In this inflammasome pathway, the activation mechanism is linked to membrane permeabilization and K+ efflux (41). To test whether potassium efflux is involved in BpOMVs-induced inflammasome activation, high extracellular concentration of K+ that inhibit its efflux from the cytosol was used to treat BMDMs. The addition of 80 mM KCl to the macrophages prior to BpOMVs stimulus reduced mIL-1β secretion (Figure 1A, *p* ≤ 0.05) without significant changes in the inflammasome-independent cytokine mTNF-α secretion (Supplementary Figure 1A), indicating that mIL-1β secretion induced by BpOMVs partially requires potassium efflux upstream NLRP3 to be activated. The induction of hIL-1β and hIL-18 secretion triggered by BpOMVs was also detected in human macrophages cells (THP1). Whereas, LPS_*E*.*coli*_ stimulates hIL-8 secretion at the same level of BpOMVs, it is no able to induce hIL-1β and hIL-18 secretion (Figure 1B, *p* ≤ 0.05). Inflammasome activation is considered to be a two-step process: priming and activation (42). To evaluate the role of MyD88 [a canonical adaptor of inflammatory signaling pathways downstream members of the Toll-like receptor (TLRs) family] in priming, THP1 XBlue™ MyD88Def cells were stimulated with BpOMVs. hIL1-β and hIL-8 secretion was completely abolished in Myd88 deficient cells (Figure 1C, *p* ≤ 0.05). SEAP (secreted alkaline phosphatase) activity in supernatants was determined as a control of NF-κB- and AP-1 stimulation in THP1-XBlue™-defMyD cells, indicating the incapacity of BpOMVs to trigger these pathways in this cell line (Supplementary Figure 1B). Conversely, hTNFα treatment used as control could induce hIL-8 and SEAP secretion (Supplementary Figure 1B). Canonical inflammasomes, such as NLRP3, are composed of a sensor protein that can recruit caspase-1 activating machinery, usually via the adaptor molecule ASC [Apoptosis-associated speck-like protein containing a caspase recruitment domain (CARD)]. During inflammasome activation, spatial re-localization of ASC into punctate structures has been described in response to stimuli (43, 44). To evaluate if BpOMVs promote ASC speck formation, THP1-ASC-GFP cells were stimulated with BpOMVs. The number of specks in BpOMVs-treated cells was significantly (*p* ≤ 0.05) higher than NTC under same conditions (Figure 2A), indicating that BpOMVs can trigger ASC complex assembly. The relation of GFP-ASC-speck-positive to total cells triggered by BpOMVs was similar to positive control condition (Ctrl+: LPS_*E*.*coli*_ primed and transfected with pcDNA as suggested by manufacturer). Representative pictures are shown with white arrowheads indicating GFP-specks (Figure 2B). hIL1-β, hIL-18, and hIL-8 secretion in THP1-ASC-GFP cells was stimulated by BpOMVs (Figure 2C, *p* ≤ 0.05). Together, these results showed that for BpOMVs-dependent inflammasome activation, features such as potassium efflux and ASC speck formation in mice BMDMs and in a human monocytic cell line (respectively) are involved. Moreover, we confirmed that the first of the two-steps inflammasome activation model depends on Myd88 adaptor.

### BpOMVs-Induced IL-1β Secretion Requires Caspase-1/11 and NLRP3

**Figure 3:**
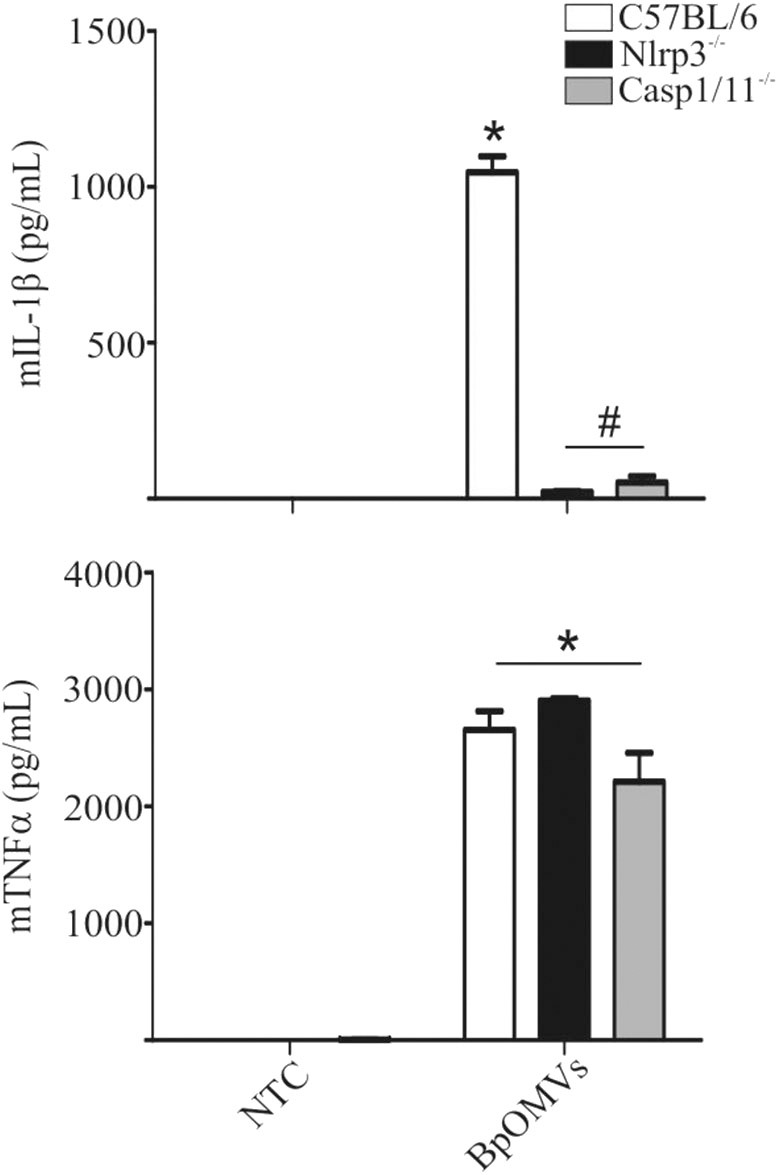
IL-1β secretion triggered by BpOMVs is NLRP3 and caspase-1/11 dependent. BMDM from C57BL/6, NLRP3^−/−^, and Casp1/11^−/−^ mice were stimulated ON with BpOMVs and mIL-1β and mTNFα were measured in supernatants. Data show the mean ± SEM from triplicate wells. *A result significantly different (*p* ≤ 0.05) than levels of untreated cells; ^#^a result significantly different from BpOMVs stimulated C57BL/6 BMDMs.

As mentioned, ASC is an adaptor protein for NLRP3 inflammasome (and several other sensors), associated to canonical activation that bridges different signals to caspase-1 activation and IL-1β cleavage (40, 45–47). We decided to assess whether NLRP3 is involved in BpOMVs-triggered IL-1β secretion. To this aim, C57BL/6, Nlrp3^−/−^, and Casp1/11^−/−^ BMDMs were stimulated with BpOMVs and IL-1β secretion was evaluated. We observed that IL-1β secretion induced by BpOMVs was completely abolished in Nlrp3^−/−^ and caspase1/11^−/−^ macrophages, whereas mTNF-α production was unaffected (Figure 3). Additionally, we stimulated C57BL/6, Nlrp3^−/−^, and Casp1/11^−/−^ BMDMs with BpOMVs and after ON stimulation, cell supernatants were collected and subjected to western blotting using specific Ab against the p20 subunit of caspase-1 and the p17 subunit of IL-1β. We observed that Nlrp3-deficient BMDMs showed a completely loss of caspase-1 and IL-1β activation and secretion in comparison to C57BL/6 macrophages which were fully able to activate caspase-1 and to process IL-1β. As a control, we stimulated Casp1/11^−/−^ which did not express caspase-1. In the case of KCl pretreatment of C57BL/6 macrophages stimulated with BpOMVs (described in the previous section), we observed reduced levels of caspase-1 and IL-1β activation and secretion in comparison to stimulated BMDM without pretreatment (Supplementary Figure 2). This result shows a similar pattern to that observed in cytokine secretion levels. Together, these results indicate that BpOMVs trigger a NLRP3 and caspase-1/11 dependent inflammasome activation.

### The Pore-Forming Protein Gasdermin-D and caspase-11 Are Involved in Non-canonical Inflammasome Activation by BpOMVs

**Figure 4:**
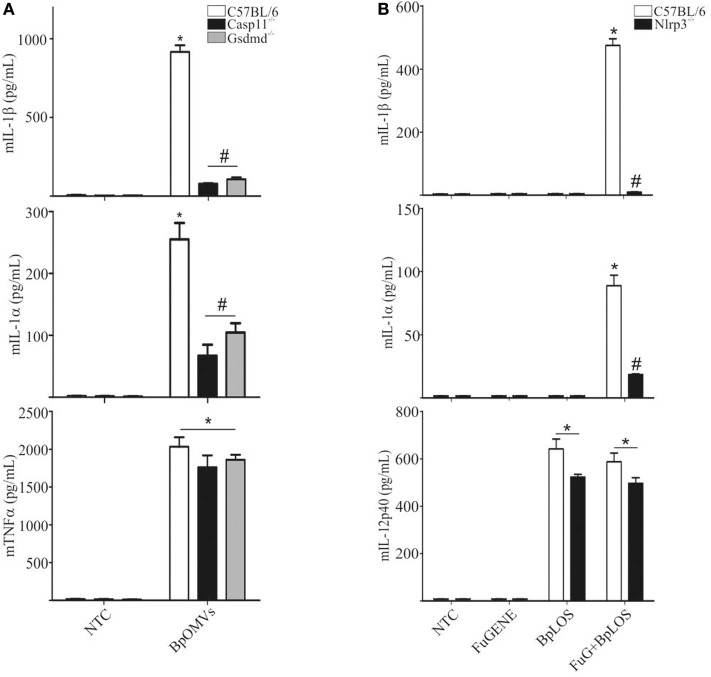
The pore-forming protein Gasdermin D and caspase-11 are involved in inflammasome activation by BpOMVs. **(A)** BMDMs from C57BL/6, Casp11^−/−^, and Gsdmd^−/−^ mice were stimulated ON with BpOMVs and mIL-1β, mIL-1α, and mTNFα were measured in supernatants by ELISA. **(B)** BMDM from C57BL/6 were stimulated with free or transfected BpLOS and mIL-1β, mIL-1α, and mIL-12 were measured in supernatants. Data are represented as mean ± SEM of three replicates. *A result significantly different (*p* ≤ 0.05) from untreated or FuGENE only treated cells; ^#^a result significantly different from BpOMVs stimulated C57BL/6 BMDMs.

The “non-canonical” inflammasome activation has been described as caspase-11 dependent (48). Caspase-11 directly interacts with cytosolic LPS resulting in cell death and inflammatory responses that also involves IL-1α (49). Caspase-1 cleaves pro-IL-1β and pro-IL-18 into its mature forms and also cleaves gasdermin-D (GSDMD) to generate a pore-forming fragment that targets the plasma membrane, while caspase-11 cleaves and activates GSDMD but cannot process cytokines like pro-IL-1β and pro-IL-18 efficiently (34, 50). As IL-1β secretion was totally abolished in Casp1/11^−/−^ BMDM stimulated with BpOMVs and it has been described that BpLOS is one of the main components of BpOMVs (28), we wondered if non-canonical inflammasome pathway was also involved in this process. To address this question, Gsdmd^−/−^ and Casp11^−/−^ BMDMs were stimulated with 800 ng/mL BpOMVs and mIL-1β and mIL-1α secretion was measured. While mTNF-α secretion was unaffected, mIL-1β and mIL-1α were completely abolished in Gsdmd^−/−^ and Casp11^−/−^ macrophages (Figure 4A, *p* ≤ 0.05). Processed IL-1β and Caspase-1 analyzed by western blot in Casp11^−/−^ BMDM supernatants were also abolished or diminished (respectively) in comparison to wild type cells (Supplementary Figure 2).

LPS chemical structure can be described into three parts: the most conserved lipid A moiety; a core oligosaccharide chain; and a variable polysaccharide chain known as “O-antigen.” With structural differences respect to other species, *B. pertussis* has a penta-acylated lipid A lipooligosaccharide that lacks an O-side chain, having in its place a non-repeating trisaccharide (51). LOS *per se* from other pathogens are able to be considered as key component responsible for both priming and licensing of inflammasome activation inducing caspase-1 activation (52). In this context, we decided to determine the contribution of BpLOS present in BpOMVs in inflammasome activation. C57BL/6 primary BMDMs produced higher levels of mIL-1β and mIL-1α in response to transfected BpLOS; however, it does not stimulate cytokine secretion when treated with free BpLOS. mIL-12 production was similar in both free and transfected BpLOS treatment (Figure 4B, *p* ≤ 0.05). Interestingly, BpLOS within the OMV is critical for triggering cytosolic sensors and inflammasome activation. Therefore, we determined whether changes in BpLOS acylation degree might influence inflammasome activation by using BpOMVs obtained from a mutant *B. pertussis* strain in LOS biosynthesis (BpOMVsPagL). This mutant strain harbors the *pagL* gene from *B. bronchiseptica*, which encodes a lipid A 3-deacylase, resulting in tetra- instead of penta-acylated BpLOS on the BpOMVs (53, 54). We confirmed that changes in BpLOS acylation influences inflammasome activation since BpOMVs_PagL_ induced a lower amount of mIL-1β and mIL-1α than wild type BpOMVs (Supplementary Figure 3). Simultaneously, Nlrp3^−/−^ BMDM were also stimulated with BpOMVs and BpOMVs_PagL_ as control, showing increased secretion of mIL-12 and impaired secretion of mIL-1β and mIL-1α (Supplementary Figure 3). Similar results have been described for tetra-acylated LPS from other gram-negative bacteria such as *F. novicida* and *Y. pestis* (55, 56), indicating that caspase-11 specificity for LPS depends on LPS acylation degree. These results indicate that caspase-11 is required for non-canonical inflammasome activation by BpOMVs, and that penta-acylated BpLOS is a key signal to trigger this response.

### NLRP3, Caspase-1/11, and GSDMD Are Involved in Inflammasome Activation by Cytoplasmatic LOS From *B. pertussis*

**Figure 5:**
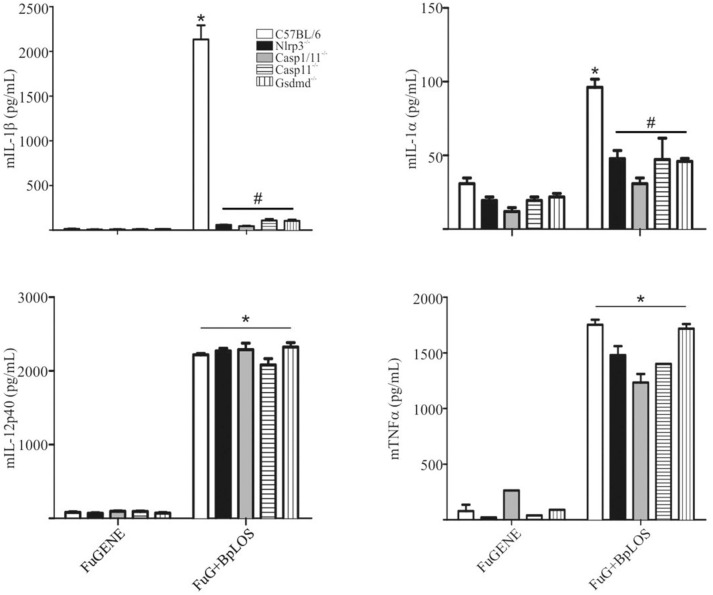
Cytosolic BpLOS contributes to non-canonical inflammasome activation. BMDM from C57BL/6, NLRP3^−/−^, Casp1/11^−/−^, Casp11^−/−^, and Gsdmd^−/−^ mice were stimulated ON with transfected BpLOS and mIL-1β, mIL-1α, mIL12p40, and mTNFα were measured in supernatants. Data are represented as mean ± SEM of three replicates. *A result significantly different (*p* ≤ 0.05) from FuGENE only treated cells; ^#^a result significantly different from FuGENE + BpLOS stimulated C57BL/6 BMDMs.

In order to confirm the direct contribution of the cytosolic BpLOS in non-canonical inflammasome activation, C57BL/6, Nlrp3^−/−^, Casp1/11^−/−^, Casp11^−/−^, and Gsdmd^−/−^ BMDMs were transfected with BpLOS. mIL-1β and mIL-1α secretion was significantly diminished in all cases compared to C57BL/6 (Figure 5, *p* ≤ 0.05, upper panels), while levels of mIL-12 and mTNFα were equivalent in all treatments (Figure 5, *p* ≤ 0.05, lower panels). So far, these results indicate that BpOMVs can activate caspase-11 and that LOS-derived from outer membrane vesicles that reach the cytosol upon endocytosis, is an important molecular signal that triggers inflammasome activation. Taken together these results show that BpLOS from BpOMVs is at least one of the components involved in inflammasome activation and that this process is dependent on NLRP3, caspase-1, caspase-11, and GSDMD.

### Guanylate-Binding Proteins Expression Is Induced by BpOMVs and BpLOS

**Figure 6:**
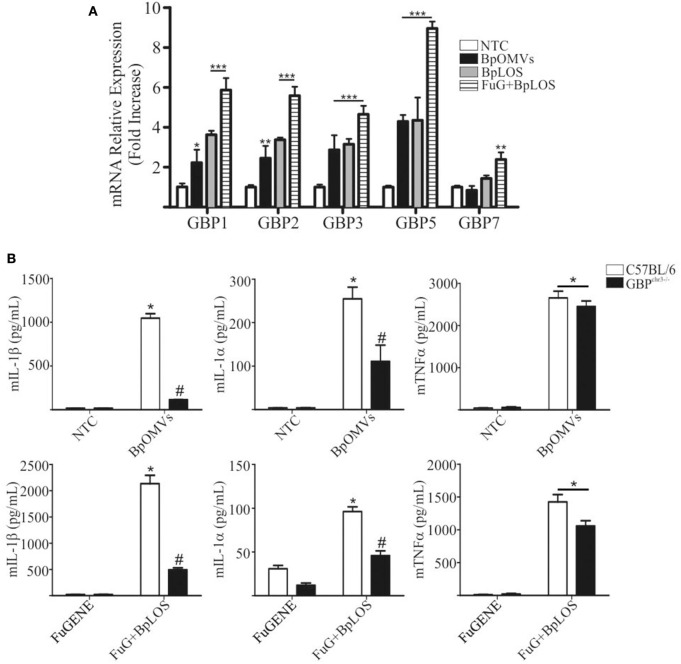
GBPs from chromosome 3 are involved in inflammasome activation triggered by BpOMVs and transfected BpLOS. **(A)** BMDMs from C57BL/6 mice were stimulated ON with BpOMVs and with free or transfected BpLOS and expression levels of GBP1, GBP2, GBP3, GBP5, and GBP7 mRNA were measured. Data are represented as mean ± SEM of three replicates. *, **, and *** represent a result significantly different 0.05, *p* ≤ 0.01 and *p* ≤ 0.001, respectively from NTC. **(B)** BMDM from C57BL/6 and GBP^*chr*3−/−^ mice were stimulated ON with BpOMVs or transfected BpLOS and mIL-1β mIL-1α and mTNFα were measured in supernatants. *represents a result significantly different (*p* ≤ 0.05) from untreated or FuGENE only treated cells; # a result significantly different from BpOMVs stimulated or BpLOS transfected C57BL/6 BMDMs.

Previous reports suggest that caspase-11 activation during LPS transfection requires Guanylate-binding proteins (GBPs) family members, and that GBP2, in particular, play an important role in this process (20, 57). The chromosomal deletion in GBPchr3 KO mice inactivates 5 GBP genes, namely Gbp1, Gbp2, Gbp3, Gbp5, and Gbp7. Cells lacking Gbpchr3 exhibit a retarded caspase-11 activation and lower levels of LPS–caspase-11 interaction, suggesting that they modulate the access of caspase-11 to LPS (57, 58). In order to determine a possible role for GBPs in BpOMVs inflammasome activation, C57BL/6 BMDMs were transfected with BpLOS or stimulated with BpOMVs or free BpLOS, and GBPs mRNA expression levels were compared. GBP1, GBP2, GBP3, and GBP5 mRNA expression levels increased in all treatment but were higher when BpLOS was transfected with FuGENE into cells. GBP7 mRNA expression levels increased exclusively with transfected BpLOS (Figure 6A). Furthermore, C57BL/6 and GBPchr3 KO BMDMs were stimulated with BpOMVs or transfected BpLOS to evaluate inflammasome activation. IL-1β and IL-1α secretion induced by BpOMVs or transfected BpLOS was diminished in Gbpchr3^−/−^ macrophages while mTNFα levels were equivalent (Figure 6B, *p* ≤ 0.05). Same results were observed for mIL-12 levels between both types of cells (Supplementary Figure 4). In addition, processed IL-1β and Caspase-1 analyzed by western blot in GBPchr3^−/−^ BMDM supernatants were also abolished or diminished (respectively) in comparison to wild type cells (Supplementary Figure 2). These observations demonstrate that GBPs play an important role in the induction of inflammasome activation by BpOMVs, possibly by facilitating the accessibility of BpLOS to cytosolic sensors such as caspase-11.

## Discussion

The importance of inflammasomes in host defense is well-established, acting as critical innate immune components that orchestrate host immune homeostasis (59, 60). Inflammasome activation is described as a two-step procedure: first, it must be primed, and then it can be activated (42). The expression of the inflammasome components NLRP3, caspase-1, and pro-IL-1β is upregulated after priming and this transcriptional upregulation can be induced through the recognition of various PAMPs or DAMPs that engage receptors such as TLRs or NOD2 or through TNF-α cytokines that lead to NF-κB activation and gene transcription (40). MyD88 adaptor is implicated in the signaling downstream of TLRs and overexpression of MyD88 leads to spontaneous activation of NFκB (61, 62). In this study, we have shown that BpOMVs are capable to induce inflammasome priming and activation of human and murine macrophages giving the increased levels of IL-1β cytokine secretion observed. Even in the absence of LPS_*E*.*coli*_ as priming stimulus, BpOMVs are able to induce the secretion of IL-1β. Furthermore, we observed that this effect is partially blocked by inhibition of K+ efflux, indicating the participation of NLRP3 inflammasome, a signaling platform that can be activated by multiple upstream events including ions efflux such as K^+^, Cl^−^, Ca^2+^, lysosomal disruption, mitochondrial dysfunction, metabolic changes, and trans-Golgi disassembly (41). NLRP3 inflammasome assembly leads to caspase-1-dependent release of the pro-inflammatory cytokines IL-1β and IL-18, as well as to gasdermin-D-mediated membrane permeabilization and pyroptotic cell death (19). This study was focused on viable cells that release IL-1β along with other proinflammatory cytokines, thus the process of pyroptosis may have a marginal contribution. The secretion of IL-1β is an unequivocal signal of inflammasome activation and in this work IL-1β detection by western blot and ELISA techniques confirmed the presence of mature IL-1β in cell supernatants following BpOMVs treatment. Inflammasome activation was also visually confirmed by fluorescent GFP-ASC “speck” oligomerization in macrophages, a hallmark of canonical inflammasome assembly. A recent study demonstrated that OMVs from EHEC (enterohemorrhagic *E. coli*) deliver LPS into the host cell cytosol by an unknown mechanism, resulting in the activation of the pro-inflammatory LPS sensor caspase-11 (7). Previously, our group has described that BpLOS in BpOMVs influences its biological activity (53). Herein, we have shown that intracellular BpLOS is sensed by caspase-11 and that BpOMVs trigger non-canonical inflammasome activation. A possible mediator of the pathogen-containing vacuoles lysis and therefore the promotion of LPS/LOS release into the cytosol are the GBPs, which facilitate the interaction of LPS with caspase-11 (58). We demonstrate that GBPs mRNA expression increases after BpOMVs and transfected BpLOS stimulation, which may indicate that GBPs play a role in the induction of inflammasome activation by BpOMVs. GBPs expression depends on signals related to interferons (57). It has been demonstrated the ability of BpOMVs to stimulate type I and II IFNs signaling pathway (63). Moreover, recent studies propose that isoprenylated GBPs can associate with the surface of OMVs or with cytosolic LPS and the factor that determines GBP recruitment is LPS itself (58). Those findings and our own results support the mechanism by which GBPs target foreign surfaces and confirm the role of GBPs in intracellular detection of LPS.

Although our findings point to LOS as an important microbial signal present in the OMVs responsible for inflammasome activation, we cannot exclude the possibility that other OMVs components may be additional signals to trigger other inflammasome pathways. It has been recently shown that different Gram-negative OMVs may vehicle bacterial DNA to host cytosol and trigger AIM2 (Absent in Melanoma 2) dependent inflammasomes (64, 65). AIM2 is an intracellular dsDNA sensor that, together with ASC, forms an inflammasome complex to activate caspase-1 and mediates the release of the proinflammatory cytokines IL-1β and IL-18 (66, 67). The AIM2 inflammasome has been reported to be essential for host-defense against several bacterial and viral pathogens (46). Furthermore, it has been reported the relevance of GBPs in the AIM2 DNA sensing during an infection model (35). Although nucleic acids seem not to be present in BpOMVs, this issue will be addressed in future work. IL-1β is required for efficient clearance of *B. pertussis* (15). The recruitment of immune cells to local sites of infection through the upregulation of chemokines and cytokines has been attributed to the activating effect of IL-1β during infection. However, studies that address the role of the inflammasome in anti-pertussis vaccination scenario are still scarce. Ag-specific Th17 and protective immunity against *B. pertussis* were demonstrated to be promoted and generated (respectively) by inflammasome mediated IL-1β (17). The authors of this work demonstrated a possible role of *B. pertussis* Adenylate Cyclase toxin in inducing the formation of an active inflammasome complex, resulting in IL-1β production. The role of IL-1β in protection against pertussis was suggested using the murine protection model (59). The authors proposed that the release of IL-1β can be initiated independently of caspase 1, which has been linked to adjuvants such as aluminum present in commercial-type vaccines (15). In the present work, we demonstrate that the BpOMVs-based vaccine platform induce IL-1 β secretion where NLRP3, caspase-1/11, and GSDMD are involved in inflammasome activation. The ability of BpOMVs to activate different inflammasome pathways can be related to the profile of immune response elicited upon vaccination. IL-1β has been suggested to promote the expansion of differentiated T cells and driving murine and human Th17 priming and phenotype stabilization (68, 69). Previously, we have demonstrated that BpOMVs based vaccine candidate promotes the induction of protective immunity against *B. pertussis* lung infection in mice. As wP vaccine, was effective at inducing *B. pertussis*-specific IFN-γ (marker of Th1 cells) and IL-17 (marker of Th17 cells) secretion by spleen cells, and Tissue-resident memory T cells (70) whereas the aP vaccine mainly induces IL-5 (marker of Th2 cells) (70, 71). In the same way, it is well-established the potency of IL-1β as an enhancer of T cell responses and the IL-1 pathway is always taken into account in the design of new adjuvants. Taken together the results of this study support a model in which BpLOS from BpOMVs is sensed by caspase-11 somehow in collaboration with GBPs from chromosome 3, leading to GSDMD processing and membrane pore formation. This membrane permeabilization enables potassium efflux and consequently, lowered intracellular potassium concentration is sensed by NLRP3 causing its complexification with ASC adaptor protein. Caspase-1 is finally activated, which in turn cleaves pro-IL-1β and more GSDMD, triggering IL-1β secretion and a new loop of activation. The results presented here showed the intrinsic capacity of BpOMVs to trigger one central pathway of innate response activation that is expected to skew the adaptive immune response elicited by BpOMVs vaccination.

## Data Availability Statement

The datasets presented in this study can be found in online repositories. The names of the repository/repositories and accession number(s) can be found in the article/Supplementary Material.

## Ethics Statement

This animal study was reviewed and approved by Committee for Ethics in Animal Experimentation—CETEA—at Universidade Federal de Minas Gerais UFMG under permit #165/2019 and Institutional Committee for the Care and Use of Laboratory Animals—CICUAL—at Universidad Nacional de La Plata approved protocol #005-06-15 extended in validity until August 10, 2023.

## Author Contributions

ME, GM, MR, DH, and SO devised the project and the main conceptual ideas. ME, MG, and EG designed and carried out the experiments. ME and MG analyzed the data and prepared the figures. GM reviewed and submitted the manuscript, and wrote the manuscript with the input of all authors. MR, DH, and SO provided the funding acquisition and supervised the project. All authors discussed the results and contributed to the final manuscript.

## Conflict of Interest

The authors declare that the research was conducted in the absence of any commercial or financial relationships that could be construed as a potential conflict of interest.

## Supplementary Material

Supplementary Figure 1 OMVs derived from *B. pertussis* (BpOMVs) triggers IL-1β in primed and unprimed murine and human macrophages. **(A)** BMDM from C57BL/6 mice were primed or not with LPS_*E*.*coli*_ for 3 h and prior to ON BpOMVs stimulation, cells were treated or not with 80 mM KCl for 30 min. mIL-1β, mTNFα, and mIL-6 were measured in supernatants. LPS_E.*coli*_ plus Nigericin was used as a positive control. ^*^A result significantly different (*p* ≤ 0.05) from NTC; ^#^a result significantly different from BpOMVs stimulation (*p* ≤ 0.05). **(B)** THP1-XBlue™-defMyD cells were stimulated with BpOMVs or TNFα (as positive control) and SEAP activity was measured in supernatants indicating MyD88-independent signaling activation. ^*^A result significantly different (*p* ≤ 0.05) from NTC.

Supplementary Figure 2 BpOMVs trigger caspase-1 and IL-1β maturation. Cell supernatants from C57BL/6 treated or not with 80 mM KCl, NLRP3^−/−^, Casp1/11^−/−^ Casp11^−/−^, and GBPchr3^−/−^ BMDMs were separated by SDS-PAGE, blotted, and probed with an anti-caspase-1 p20 subunit and anti-IL-1β p17 subunit monoclonal antibody. As loading control, cell lysates were probed with anti-β-actin monoclonal antibody.

Supplementary Figure 3 BpLOS acylation influences inflammasome activation. BMDM from C57BL/6 were stimulated ON with BpOMVs or LOS-modified BpOMVsPagL and mIL-1β, mIL1-α, and mIL-12p40 were measured in supernatants. ^*^A result significantly different (*p* ≤ 0.05) from NTC; ^#^a result significantly different from the wild type cells under similar stimulation (*p* ≤ 0.05); a result significantly different from BpOMVs stimulation (*p* ≤ 0.05).

Supplementary Figure 4 GBPs from chromosome 3 are involved in inflammasome activation triggered by BpOMVs and transfected BpLOS. C57BL/6 and GBPchr3^−/−^ BMDMs were stimulated with BpOMVs or transfected BpLOS and mIL-12 levels were measured in supernatants. ^*^A result significantly different (*p* ≤ 0.05) from NTC or to transfection reagent FuGene treatment, respectively.

## References

[1] Amano A Takeuchi H Furuta N. Outer membrane vesicles function as offensive weapons in host-parasite interactions. Microbes Infect. (2010) 12:791–8. 10.1016/j.micinf.2010.05.008 20685339

[2] Bonnington KE Kuehn MJ. Protein selection and export via outer membrane vesicles. Biochim Biophys Acta. (2014) 1843:1612–9. 10.1016/j.bbamcr.2013.12.011 24370777 PMC4317292

[3] Alaniz RC Deatherage BL Lara JC Cookson BT. Membrane vesicles are immunogenic facsimiles of salmonella typhimurium that potently activate dendritic cells, prime B and T cell responses, and stimulate protective immunity *in vivo*. J Immunol. (2007) 179:7692–701. 10.4049/jimmunol.179.11.7692 18025215

[4] Lindmark B Rompikuntal PK Vaitkevicius K Song T Mizunoe Y Uhlin BE. Outer membrane vesicle-mediated release of cytolethal distending toxin (CDT) from campylobacter jejuni. BMC Microbiol. (2009) 9:220. 10.1186/1471-2180-9-220 19835618 PMC2770062

[5] Renelli M Matias V Lo RY Beveridge TJ. DNA-containing membrane vesicles of pseudomonas aeruginosa PAO1 and their genetic transformation potential. Microbiology. (2004) 150:2161–9. 10.1099/mic.0.26841-0 15256559

[6] Grubman A Kaparakis M Viala J Allison C Badea L Karrar A. The innate immune molecule, NOD1, regulates direct killing of helicobacter pylori by antimicrobial peptides. Cell Microbiol. (2010) 12:626–39. 10.1111/j.1462-5822.2009.01421.x 20039881

[7] Vanaja SK Russo AJ Behl B Banerjee I Yankova M Deshmukh SD. Bacterial outer membrane vesicles mediate cytosolic localization of lps and caspase-11 activation. Cell. (2016) 165:1106–19. 10.1016/j.cell.2016.04.015 27156449 PMC4874922

[8] Koeppen K Hampton TH Jarek M Scharfe M Gerber SA Mielcarz DW. A novel mechanism of host-pathogen interaction through srna in bacterial outer membrane vesicles. PLoS Pathog. (2016) 12:E1005672. 10.1371/journal.ppat.1005672 27295279 PMC4905634

[9] Ellis TN Kuehn MJ. Virulence and immunomodulatory roles of bacterial outer membrane vesicles. Microbiol Mol Biol Rev. (2010) 74:81–94. 10.1128/MMBR.00031-09 20197500 PMC2832350

[10] Pathirana RD Kaparakis-Liaskos M. Bacterial membrane vesicles: biogenesis, immune regulation and pathogenesis. Cell Microbiol. (2016) 12:1518–24. 10.1111/cmi.12658 27564529

[11] Cai W Kesavan DK Wan J Abdelaziz MH Su Z Xu H. Bacterial outer membrane vesicles, a potential vaccine candidate in interactions with host cells based. Diagn Pathol. (2018) 13:95. 10.1186/s13000-018-0768-y 30537996 PMC6290530

[12] Hozbor DF. Outer membrane vesicles: an attractive candidate for pertussis vaccines. Expert Rev Vaccines. (2017) 16:193–6. 10.1080/14760584.2017.1276832 28010142

[13] Erlich Z Shlomovitz I Edry-Botzer L Cohen H Frank D Wang H. Macrophages, rather than DCs, are responsible for inflammasome activity in the GM-CSF BMDC model. Nat Immunol. (2019) 20:397–406. 10.1038/s41590-019-0313-5 30742078

[14] Kroes MM Mariman R Hijdra D Hamstra H-J van Boxtel KJWM van Putten JPM. Activation of human NK cells by bordetella pertussis requires inflammasome activation in macrophages. Front Immunol. (2019) 10:2030. 10.3389/fimmu.2019.02030 31507615 PMC6718514

[15] Place DE Muse SJ Kirimanjeswara GS Harvill ET. Caspase-1-independent interleukin-1β is required for clearance of bordetella pertussis infections and whole-cell vaccine-mediated immunity. PLoS ONE. (2014) 9:e107188. 10.1371/journal.pone.0107188 25198773 PMC4157866

[16] Evavold CL Kagan JC. How inflammasomes inform adaptive immunity. J Mol Biol. (2018) 430:217–37. 10.1016/j.jmb.2017.09.019 28987733 PMC5766381

[17] Dunne A Ross PJ Pospisilova E Masin J Meaney A Sutton CE. Inflammasome activation by adenylate cyclase toxin directs Th17 responses and protection against bordetella pertussis. J Immunol. (2010) 185:1711–9. 10.4049/jimmunol.1000105 20610650

[18] Broz P Pelegrín P Shao F. The gasdermins, a protein family executing cell death and inflammation. Nat Rev Immunol. (2019) 20:143–57. 10.1038/s41577-019-0228-2 31690840

[19] Chen KW Monteleone M Boucher D Sollberger G Ramnath D Condon ND. Noncanonical inflammasome signaling elicits gasdermin D–dependent neutrophil extracellular traps. Sci Immunol. (2018) 3:eaar6676. 10.1126/sciimmunol.aar6676 30143554

[20] Gomes MTR Cerqueira DM Guimarães ES Campos PC Oliveira SC. Guanylate-binding proteins at the crossroad of noncanonical inflammasome activation during bacterial infections. J Leukoc Biol. (2019) 106:553–62. 10.1002/JLB.4MR0119-013R 30897250 PMC7516346

[21] Uchiyama R Yonehara S Taniguchi S Ishido S Ishii KJ Tsutsui H. Inflammasome and fas-mediated IL-1β contributes to Th17/Th1 cell induction in pathogenic bacterial infection *in vivo*. J Immunol. (2017) 199:1122–30. 10.4049/jimmunol.1601373 28674179

[22] Libster R Edwards KM. Re-emergence of pertussis: what are the solutions? Expert Rev Vaccines. (2012) 11:1331–46. 10.1586/erv.12.118 23249233

[23] Chang IF Lee PI Lu CY Chen JM Huang LM Chang LY. Resurgence of pertussis in Taiwan during 2009–2015 and its impact on infants. J Microbiol Immunol Infect. (2019) 52:542–8. 10.1016/j.jmii.2019.06.002 31285158

[24] Tan T Dalby T Forsyth K Halperin SA Heininger U Hozbor D. Pertussis across the globe: recent epidemiologic trends from 2000 to 2013. Pediatr Infect Dis J. (2015) 34:e222–32. 10.1097/INF.0000000000000795 26376316

[25] Mooi FR Van Der Maas NAT De Melker HE. Pertussis resurgence: waning immunity and pathogen adaptation - two sides of the same coin. Epidemiol Infect. (2014) 142:685–94. 10.1017/S0950268813000071 23406868 PMC9151166

[26] McGirr A Fisman DN. Duration of pertussis immunity after DTaP immunization: a meta-analysis. Pediatrics. (2015) 135:331–43. 10.1542/peds.2014-1729 25560446

[27] Bart MJ Harris SR Advani A Arakawa Y Bottero D Bouchez V. Global population structure and evolution of bordetella pertussis and their relationship with vaccination. MBio. (2014) 5:14. 10.1128/mBio.01074-14 24757216 PMC3994516

[28] Roberts R Moreno G Bottero D Gaillard ME Fingermann M Graieb A. Outer membrane vesicles as acellular vaccine against pertussis. Vaccine. (2008) 26:4639–46. 10.1016/j.vaccine.2008.07.004 18640169

[29] Rumbo M Hozbor D. Development of improved pertussis vaccine. Hum Vaccin Immunother. (2014) 10:2450–3. 10.4161/hv.29253 25424954 PMC4896757

[30] Hozbor D Rodriguez ME Fernández J Lagares A Guiso N Yantorno O. Release of outer membrane vesicles from bordetella pertussis. Curr Microbiol. (1999) 38:273–8. 10.1007/PL00006801 10355115

[31] Mariathasan S Weiss DS Newton K McBride J O'Rourke K Roose-Girma M. Cryopyrin activates the inflammasome in response to toxins and ATP. Nature. (2006) 440:228–32. 10.1038/nature04515 16407890

[32] Kuida K Lippke JA Ku G Harding MW Livingston DJ Su MSS. Altered cytokine export and apoptosis in mice deficient in interleukin-1β converting enzyme. Science. (1995) 267:2000–3. 10.1126/science.7535475 7535475

[33] Kayagaki N Warming S Lamkanfi M Walle L. Vande Louie S Dong J. Non-canonical inflammasome activation targets caspase-11. Nature. (2011) 479:117–21. 10.1038/nature10558 22002608

[34] Kayagaki N Stowe IB Lee BL O'Rourke K Anderson K Warming S. Caspase-11 cleaves gasdermin D for non-canonical inflammasome signalling. Nature. (2015) 526:666–71. 10.1038/nature15541 26375259

[35] Man SM Karki R Malireddi RKS Neale G Vogel P Yamamoto M. The transcription factor IRF1 and guanylate-binding proteins target activation of the AIM2 inflammasome by francisella infection. Nat Immunol. (2015) 16:467–75. 10.1038/ni.3118 25774715 PMC4406811

[36] Yamamoto M Okuyama M Ma JS Kimura T Kamiyama N Saiga H. A cluster of interferon-γ-inducible p65 gtpases plays a critical role in host defense against toxoplasma gondii. Immunity. (2012) 37:302–13. 10.1016/j.immuni.2012.06.009 22795875

[37] Degrandi D Kravets E Konermann C Beuter-Gunia C Klumpers V Lahme S. Murine guanylate binding protein 2 (mGBP2) controls toxoplasma gondii replication. Proc Natl Acad Sci USA. (2013) 110:294–9. 10.1073/pnas.1205635110 23248289 PMC3538222

[38] Weischenfeldt J Porse B. Bone marrow-derived macrophages (BMM): isolation and applications. Cold Spring Harb Protoc. (2008) 2008:pdb.prot5080. 10.1101/pdb.prot5080 21356739

[39] Zurita E Moreno G Errea A Ormazabal M Rumbo M Hozbor D. The stimulated innate resistance event in bordetella pertussis infection is dependent on reactive oxygen species production. Infect Immun. (2013) 81:2371–8. 10.1128/IAI.00336-13 23630952 PMC3697608

[40] Swanson KV Deng M Ting JP-Y. The NLRP3 inflammasome: molecular activation and regulation to therapeutics. Nat Rev Immunol. (2019) 19:477–89. 10.1038/s41577-019-0165-0 31036962 PMC7807242

[41] Muñoz-Planillo R Kuffa P Martínez-Colón G Smith BL Rajendiran TM Núñez G. K+ Efflux Is the common trigger of nlrp3 inflammasome activation by bacterial toxins and particulate matter. Immunity. (2013) 38:1142–53. 10.1016/j.immuni.2013.05.016 23809161 PMC3730833

[42] Horvath GL Schrum JE de Nardo CM Latz E. Intracellular sensing of microbes and danger signals by the inflammasomes. Immunol Rev. (2011) 243:119–35. 10.1111/j.1600-065X.2011.01050.x 21884172 PMC3893570

[43] Stutz A Horvath GL Monks BG Latz E. ASC speck formation as a readout for inflammasome activation. Methods Mol Biol. (2013) 1040:91–101. 10.1007/978-1-62703-523-1_8 23852599

[44] Lage SL Dominical VM Wong CS Sereti I. Evaluation of canonical inflammasome activation in human monocytes by imaging flow cytometry. Front Immunol. (2019) 10:1284. 10.3389/fimmu.2019.01284 31214205 PMC6558012

[45] Franchi L Kamada N Nakamura Y Burberry A Kuffa P Suzuki S. NLRC4-driven production of IL-1β discriminates between pathogenic and commensal bacteria and promotes host intestinal defense. Nat Immunol. (2012) 13:449–6. 10.1038/ni.2263 22484733 PMC3361590

[46] Rathinam VAK Jiang Z Waggoner SN Sharma S Cole LE Waggoner L. The AIM2 inflammasome is essential for host defense against cytosolic bacteria and DNA viruses. Nat Immunol. (2010) 11:395–402. 10.1038/ni.1864 20351692 PMC2887480

[47] Duncan JA Canna SW. The NLRC4 inflammasome. Immunol Rev. (2018) 281:115–23. 10.1111/imr.12607 29247997 PMC5897049

[48] Yi Y-S. Caspase-11 non-canonical inflammasome: a critical sensor of intracellular lipopolysaccharide in macrophage-mediated inflammatory responses. Immunology. (2017) 152:207–17. 10.1111/imm.12787 28695629 PMC5588777

[49] Yang J Zhao Y Shao F. Non-canonical activation of inflammatory caspases by cytosolic LPS in innate immunity. Curr Opin Immunol. (2015) 32:78–83. 10.1016/j.coi.2015.01.007 25621708

[50] Shi J Zhao Y Wang K Shi X Wang Y Huang H. Cleavage of GSDMD by inflammatory caspases determines pyroptotic cell death. Nature. (2015) 526:660–5. 10.1038/nature15514 26375003

[51] Fedele G Nasso M Spensieri F Palazzo R Frasca L Watanabe M. Lipopolysaccharides from bordetella pertussis and bordetella parapertussis differently modulate human dendritic cell functions resulting in divergent prevalence of Th17-polarized responses. J Immunol. (2008) 181:208–16. 10.4049/jimmunol.181.1.208 18566386

[52] Idosa BA Kelly A Jacobsson S Demirel I Fredlund H Särndahl E. Neisseria meningitidis-Induced caspase-1 activation in human innate immune cells is LOS-dependent. J Immunol Res. (2019) 2019:6193186. 10.1155/2019/6193186 31198794 PMC6526529

[53] Asensio CJA Gaillard ME Moreno G Bottero D Zurita E Rumbo M. Outer membrane vesicles obtained from bordetella pertussis tohama expressing the lipid a deacylase pagL as a novel acellular vaccine candidate. Vaccine. (2011) 29:68. 10.1016/j.vaccine.2010.12.068 21211579

[54] Geurtsen J Steeghs L Hamstra HJ Ten Hove J De Haan A Kuipers B. Expression of the lipopolysaccharide-modifying enzymes PagP and PagL modulates the endotoxic activity of bordetella pertussis. Infect Immun. (2006) 74:5574–85. 10.1128/IAI.00834-06 16988232 PMC1594925

[55] Hagar JA Powell DA Aachoui Y Ernst RK Miao EA. Cytoplasmic LPS activates caspase-11: implications in TLR4-independent endotoxic shock. Science. (2013) 341:1250–3. 10.1126/science.1240988 24031018 PMC3931427

[56] Lagrange B Benaoudia S Wallet P Magnotti F Provost A Michal F. Human caspase-4 detects tetra-acylated LPS and cytosolic francisella and functions differently from murine caspase-11. Nat Commun. (2018) 9:242. 10.1038/s41467-017-02682-y 29339744 PMC5770465

[57] Kim B-H Chee JD Bradfield CJ Park E-S Kumar P MacMicking JD. Interferon-induced guanylate-binding proteins in inflammasome activation and host defense. Nat Immunol. (2016) 17:481–9. 10.1038/ni.3440 27092805 PMC4961213

[58] Santos JC Dick MS Lagrange B Degrandi D Pfeffer K Yamamoto M LPS targets host guanylate-binding proteins to the bacterial outer membrane for non-canonical inflammasome activation. EMBO J. (2018) 7:e98089 10.15252/embj.201798089 PMC5852652 29459437

[59] Palazon-Riquelme P Lopez-Castejon G. The inflammasomes, immune guardians at defence barriers. Immunology. (2018) 155:320–30. 10.1111/imm.12989 30098204 PMC6187212

[60] Lamkanfi M Dixit VM. Mechanisms and functions of inflammasomes. Cell. (2014) 157:1013–22. 10.1016/j.cell.2014.04.007 24855941

[61] Medzhitov R Preston-Hurlburt P Kopp E Stadlen A Chen C Ghosh S. MyD88 is an adaptor protein in the hToll/IL-1 receptor family signaling pathways. Mol Cell. (1998) 2:253–8. 10.1016/S1097-2765(00)80136-7 9734363

[62] Deguine J Barton GM. MyD88: a central player in innate immune signaling. F1000Prime Rep. (2014) 6:97. 10.12703/P6-97 25580251 PMC4229726

[63] Brummelman J Raeven RHM Helm K Pennings JLA Metz B Van Eden W. Transcriptome signature for dampened Th2 dominance in acellular pertussis vaccine-induced CD4 + T cell responses through TLR4 ligation. Sci Rep. (2016) 6:25064. 10.1038/srep25064 27118638 PMC4846868

[64] Bitto NJ Chapman R Pidot S Costin A Lo C Choi J. Bacterial membrane vesicles transport their DNA cargo into host cells. Sci Rep. (2017) 7:7072. 10.1038/s41598-017-07288-4 28765539 PMC5539193

[65] Cecil JD O'Brien-Simpson NM Lenzo JC Holden JA Singleton W Perez-Gonzalez A. Outer membrane vesicles prime and activate macrophage inflammasomes and cytokine secretion *in vitro* and *in vivo*. Front Immunol. (2017) 8:1017. 10.3389/fimmu.2017.01017 28890719 PMC5574916

[66] Fernandes-Alnemri T Yu JW Datta P Wu J Alnemri ES. AIM2 activates the inflammasome and cell death in response to cytoplasmic DNA. Physiol Behav. (2009) 176:139–48. 10.1038/nature07710 19158676 PMC2862225

[67] Man SM Karki R Kanneganti TD. DNA-sensing inflammasomes: regulation of bacterial host defense and the gut microbiota. Pathog Dis. (2016) 74:ftw028. 10.1093/femspd/ftw028 27056948 PMC5985483

[68] Ben-Sasson SZ Caucheteux S Crank M Hu-Li J Paul WE. IL-1 acts on T cells to enhance the magnitude of *in vivo* immune responses. Cytokine. (2011) 56:122–5. 10.1016/j.cyto.2011.07.006 21843950 PMC3171626

[69] Ben-Sasson SZ Hu-Li J Quiel J Cauchetaux S Ratner M Shapira I. IL-1 acts directly on CD4 T cells to enhance their antigen-driven expansion and differentiation. Proc Natl Acad Sci USA. (2009) 106:7119–24. 10.1073/pnas.0902745106 19359475 PMC2678417

[70] Zurita ME Wilk MM Carriquiriborde F Bartel E Moreno G Misiak A. A pertussis outer membrane vesicle-based vaccine induces lung-resident memory CD4 T cells and protection against bordetella pertussis, including pertactin deficient strains. Front Cell Infect Microbiol. (2019) 9:125. 10.3389/fcimb.2019.00125 31106160 PMC6498398

[71] Bottero D Gaillard ME Zurita E Moreno G Martinez DS Bartel E. Characterization of the immune response induced by pertussis OMVs-based vaccine. Vaccine. (2016) 34:3303–9. 10.1016/j.vaccine.2016.04.079 27151884

