# Supplementary figures and images for: Canonical and Non-canonical Inflammasome Activation by Outer Membrane Vesicles Derived From Bordetella pertussis
Source: Front Immunol. 2020 Aug 20;11:1879. doi: 10.3389/fimmu.2020.01879 (PMC7468456; doi:10.3389/fimmu.2020.01879)

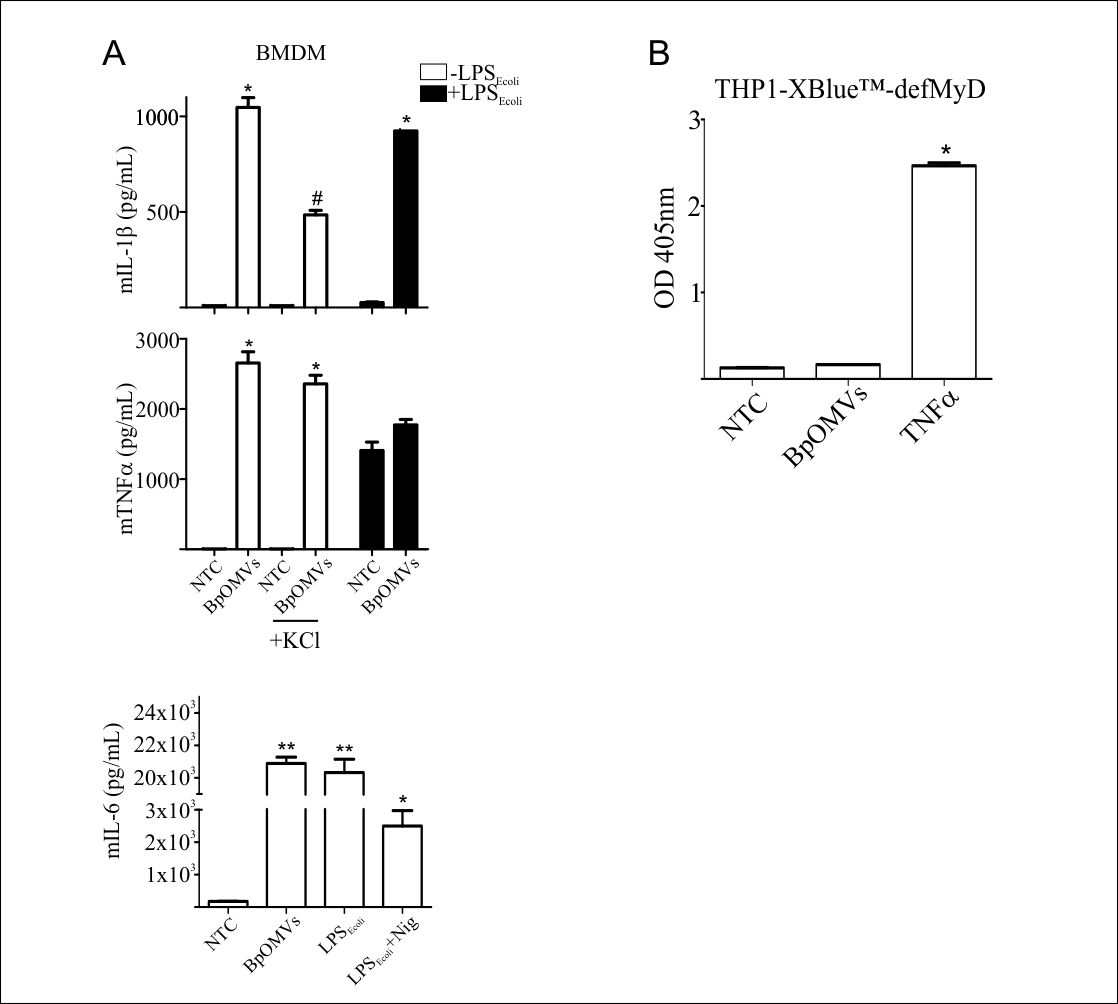

Supplement: Supplementary Figure 1 — OMVs derived from B. pertussis (BpOMVs) triggers IL-1β in primed and unprimed murine and human macrophages. (A) BMDM from C57BL/6 mice were primed or not with LPSE.coli for 3 h and prior to ON BpOMVs stimulation, cells were treated or not with 80 mM KCl for 30 min. mIL-1β, mTNFα, and mIL-6 were measured in supernatants. LPSE.coli plus Nigericin was used as a positive control. *A result significantly different (p ≤ 0.05) from NTC; #a result significantly different from BpOMVs stimulation (p ≤ 0.05). (B) THP1-XBlue™-defMyD cells were stimulated with BpOMVs or TNFα (as positive control) and SEAP activity was measured in supernatants indicating MyD88-independent signaling activation. *A result significantly different (p ≤ 0.05) from NTC. [file Image_1.jpg]

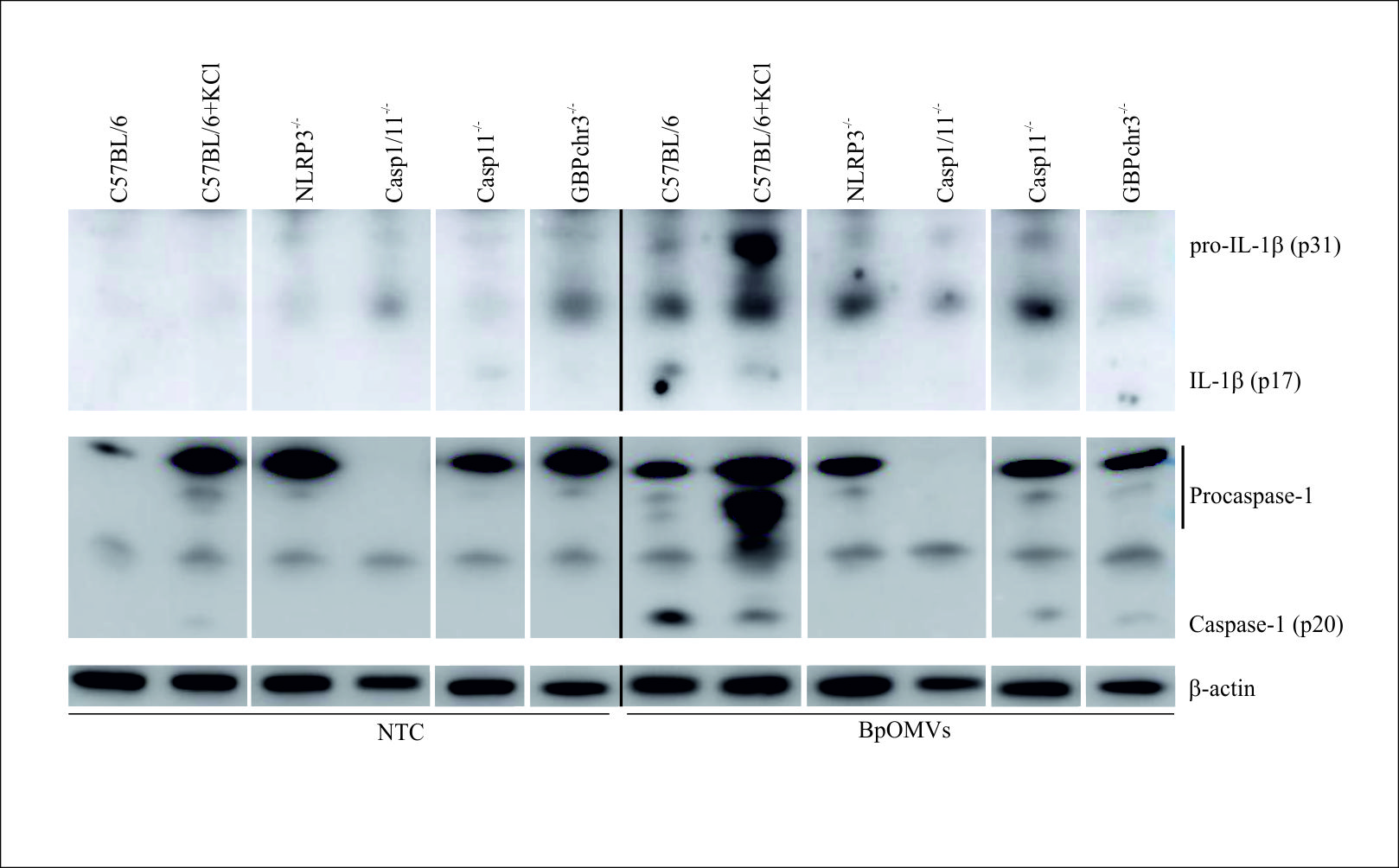

Supplement: Supplementary Figure 2 — BpOMVs trigger caspase-1 and IL-1β maturation. Cell supernatants from C57BL/6 treated or not with 80 mM KCl, NLRP3−/−, Casp1/11−/− Casp11−/−, and GBPchr3−/− BMDMs were separated by SDS-PAGE, blotted, and probed with an anti-caspase-1 p20 subunit and anti-IL-1β p17 subunit monoclonal antibody. As loading control, cell lysates were probed with anti-β-actin monoclonal antibody. [file Image_2.jpg]

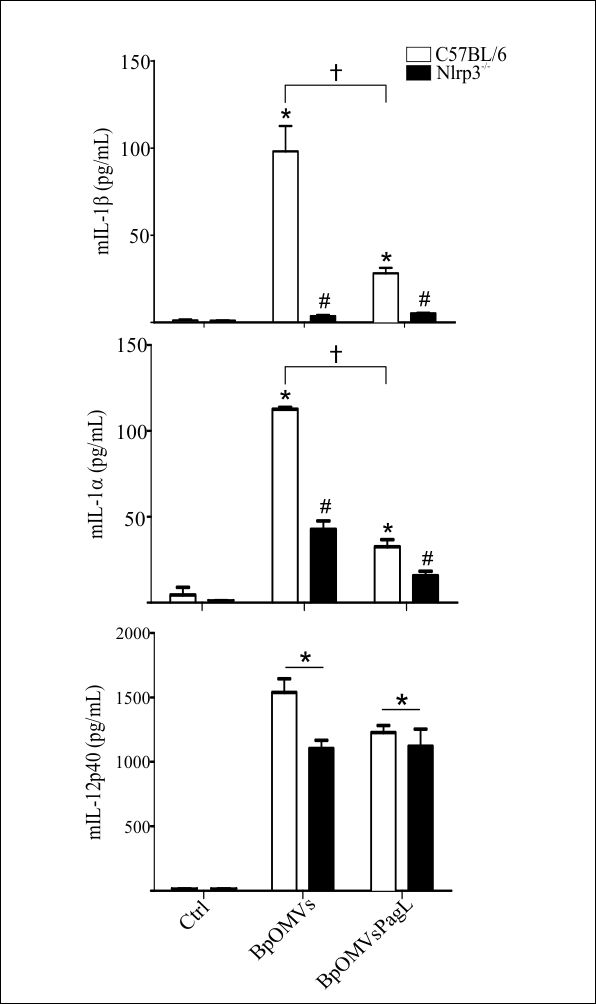

Supplement: Supplementary Figure 3 — BpLOS acylation influences inflammasome activation. BMDM from C57BL/6 were stimulated ON with BpOMVs or LOS-modified BpOMVsPagL and mIL-1β, mIL1-α, and mIL-12p40 were measured in supernatants. *A result significantly different (p ≤ 0.05) from NTC; #a result significantly different from the wild type cells under similar stimulation (p ≤ 0.05); a result significantly different from BpOMVs stimulation (p ≤ 0.05). [file Image_3.jpg]

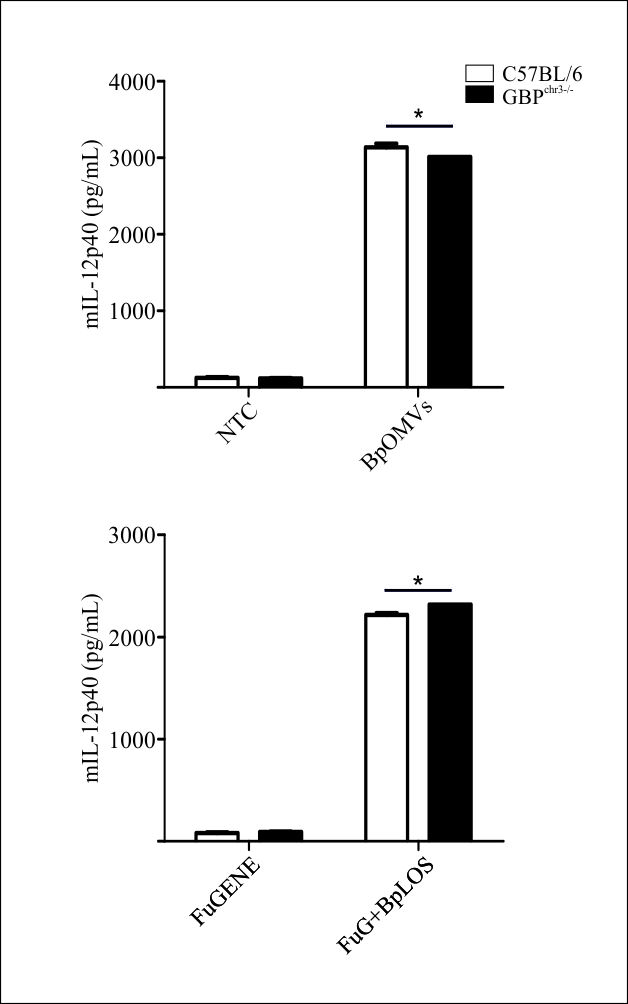

Supplement: Supplementary Figure 4 — GBPs from chromosome 3 are involved in inflammasome activation triggered by BpOMVs and transfected BpLOS. C57BL/6 and GBPchr3−/− BMDMs were stimulated with BpOMVs or transfected BpLOS and mIL-12 levels were measured in supernatants. *A result significantly different (p ≤ 0.05) from NTC or to transfection reagent FuGene treatment, respectively. [file Image_4.jpg]
